# A Systems Approach to Brain Tumor Treatment

**DOI:** 10.3390/cancers13133152

**Published:** 2021-06-24

**Authors:** James H. Park, Adrian Lopez Garcia de Lomana, Diego M. Marzese, Tiffany Juarez, Abdullah Feroze, Parvinder Hothi, Charles Cobbs, Anoop P. Patel, Santosh Kesari, Sui Huang, Nitin S. Baliga

**Affiliations:** 1 Institute for Systems Biology, Seattle, WA 98109, USA; james.park@isbscience.org (J.H.P.); sui.huang@isbscience.org (S.H.); 2 Center for Systems Biology, University of Iceland, 101 Reykjavik, Iceland; adrian@hi.is; 3 Balearic Islands Health Research Institute (IdISBa), 07010 Palma, Spain; diego.marzese@ssib.es; 4 St. John’s Cancer Institute, Santa Monica, CA 90401, USA; tiffany.juarez@providence.org (T.J.); santoshkesari@providence.org (S.K.); 5 Department of Neurological Surgery, University of Washington, Seattle, WA 98195, USA; aferoze@uw.edu (A.F.); apatel1@neurosurgery.washington.edu (A.P.P.); 6 Swedish Neuroscience Institute, Seattle, WA 98122, USA; parvinder.hothi@swedish.org (P.H.); charles.cobbs@swedish.org (C.C.); 7 Ben and Catherine Ivy Center for Advanced Brain Tumor Treatment, Seattle, WA 98122, USA; 8 Human Biology Division, Fred Hutchinson Cancer Research Center, Seattle, WA 98109, USA; 9 Brotman-Baty Institute for Precision Medicine, University of Washington, Seattle, WA 98195, USA; 10 Departments of Microbiology, Biology, and Molecular Engineering Sciences, University of Washington, Seattle, WA 98105, USA

**Keywords:** glioblastoma, brain metastases, intratumoral heterogeneity, systems biology, precision medicine

## Abstract

**Simple Summary:**

Pronounced differences across individuals (interpatient variability) and cell–cell heterogeneity within a tumor (intratumoral heterogeneity) severely hinder effective brain tumor treatment. To overcome these challenges, a personalized precision medicine approach that considers the uniqueness of an individual patient’s tumor and its cellular composition is required. A systems biology approach is needed to develop a multiscale understanding of the mechanistic drivers of disease etiology and progression to realize this vision. A systems-level understanding of disease characteristics can facilitate precise patient stratification into clinically meaningful subtypes and inform on potential druggable targets that can enhance treatment. Here, we synthesize and review various methodologies that can be integrated into a framework designed to achieve a personalized precision medicine approach for treating brain tumors. Finally, we provide a practical example in the context of analyzing an individual glioblastoma (GBM) patient at various stages of disease progression.

**Abstract:**

Brain tumors are among the most lethal tumors. Glioblastoma, the most frequent primary brain tumor in adults, has a median survival time of approximately 15 months after diagnosis or a five-year survival rate of 10%; the recurrence rate is nearly 90%. Unfortunately, this prognosis has not improved for several decades. The lack of progress in the treatment of brain tumors has been attributed to their high rate of primary therapy resistance. Challenges such as pronounced inter-patient variability, intratumoral heterogeneity, and drug delivery across the blood–brain barrier hinder progress. A comprehensive, multiscale understanding of the disease, from the molecular to the whole tumor level, is needed to address the intratumor heterogeneity resulting from the coexistence of a diversity of neoplastic and non-neoplastic cell types in the tumor tissue. By contrast, inter-patient variability must be addressed by subtyping brain tumors to stratify patients and identify the best-matched drug(s) and therapies for a particular patient or cohort of patients. Accomplishing these diverse tasks will require a new framework, one involving a systems perspective in assessing the immense complexity of brain tumors. This would in turn entail a shift in how clinical medicine interfaces with the rapidly advancing high-throughput (HTP) technologies that have enabled the omics-scale profiling of molecular features of brain tumors from the single-cell to the tissue level. However, several gaps must be closed before such a framework can fulfill the promise of precision and personalized medicine for brain tumors. Ultimately, the goal is to integrate seamlessly multiscale systems analyses of patient tumors and clinical medicine. Accomplishing this goal would facilitate the rational design of therapeutic strategies matched to the characteristics of patients and their tumors. Here, we discuss some of the technologies, methodologies, and computational tools that will facilitate the realization of this vision to practice.

## Highlights

- Brain tumors are difficult to treat because inter-patient and intratumoral heterogeneity are particularly pronounced. Moreover, the blood–brain barrier (BBB) presents an impediment to drug delivery.
- Technological advancements now allow robust profiling of genomic, epigenomic, transcriptomic, proteomic, and metabolomic changes in brain tumors.
- In parallel, efficient computational approaches to probe multi-modal data extensively and to infer regulatory mechanisms distinguishing tumor cell subpopulations have fostered novel intervention strategies (e.g., targeted- and immunotherapies) to treat brain tumors effectively.
- To address the current gaps preventing the translation of these advancements into clinical practice, a procedural framework that incorporates sensitive diagnostic and prognostic tests and computational methodologies must be developed to analyze clinical molecular profiles, enable relevant patient stratification, and identify appropriate treatments for *N*-of-1 therapy selection.

## 1. Introduction

Brain tumors contribute to tens of thousands of deaths per year, with an estimated 17,760 deaths in 2019 in the U.S.A. [1]. Glioblastoma (GBM) and brain metastasis (BM) are the most commonly diagnosed malignant brain tumors and are among the most difficult tumor types to treat. In particular, complete resection of a GBM tumor can be extremely difficult to achieve due to the highly invasive nature of GBM cells that tend to spread to the surrounding brain tissue. The current standard of care for GBM includes a combination of surgical resection, adjuvant radiotherapy, and chemotherapy (temozolomide, TMZ) [2,3]. The prognosis of GBM patients who receive the standard of care (SOC) remains dismal, with a median survival time of approximately 15 months, a five-year survival rate of less than 10%, and a recurrence rate of ~90% [4]. For BM patients, prognosis is similarly poor. The survival rate following resection and whole-brain radiation therapy ranges from three to six months [5] and the recurrence rate is as high as 76% in patients receiving a unimodal treatment [6,7]. Towards identifying the pathogenesis of brain tumors and targetable mechanisms, traditional “reductionist” approaches have generated a tremendous amount of insight and identified various mechanisms that contribute to tumor maintenance, progression, and drug resistance [8,9,10,11,12,13,14,15,16]. However, we have only seen marginal improvements in the treatment and prognosis of brain tumors over the last decade. 

The lack of clinical improvement reflects the inherent multi-scale and omic-wide complexity that is characteristic of many tumors and is particularly pronounced in brain tumors. The multitude of biological processes that drive brain tumor pathogenesis have become evident with the arrival of new omics technologies. Specifically, omics analysis in a growing number of patients has exposed the full extent of tumor heterogeneity among brain tumors. There are two main challenges to overcome: *intratumoral heterogeneity* and *inter-patient variability*. The latter is sometimes inappropriately called “inter-tumor heterogeneity” [17] because the null-model has never been one of “homogeneity”, neither of a patient cohort, nor of a tumor. What is often mistaken for “inter-tumor heterogeneity” is rather a composite problem involving the immense diversity of tumor subtypes and of individuals within a population that respond differently to the same driver mutations or treatment—hence, emphasis should be placed on ‘inter-patient’ as opposed to simply ‘inter-tumor’ variability [18]. Even if isogenic, no two individuals, let alone tumors that may arise, are identical due to distinct patient backgrounds and exposure histories. Furthermore, even if the latter were hypothetically identical, the tumor phenotypes would still be distinct due to the non-linear dynamics of molecular and cell–cell interactions and the stochastic nature of biological processes that amplify tiny differences in their initial states. These factors underlie the uniqueness of every living organism and are at the core of the difficulty in finding predictive biomarkers or drugs that have universal efficacy. Random mutations further exaggerate the uniqueness in tumors. This multi-level diversity also contributes to the multiplicity of mechanisms that confer drug resistance [14,16,19,20] and to the failure of many single-target therapies in clinical trials. 

This uniqueness spans multiple biological levels but is already quite pervasive at the single-cell level. Stochastic, complex, and non-linear biological processes that translate genomic information into a cell phenotype produce variations in the molecular state across individual cells. These variations lead to distinctive differences in their response(s) to intrinsic and extrinsic signals that trigger cell-state transitions. The result is a heterogeneous population of cells that exist in various phenotypic states, even if the population is clonal [21,22]. In the context of a tumor cell population, this heterogeneity can manifest as phenotypically distinct groups of tumor cells or subpopulations within a tumor [23]. Tumor cell heterogeneity negates the effectiveness of single-target, molecular rationale-based therapies designed from data obtained in bulk (cell population-level) analyses; the precise characteristics of a heterogeneous tumor cell population, such as the presence of biologically distinct subpopulations that would respond to different treatments, are not adequately captured by bulk-level analyses. The need to characterize and quantify subpopulations within a tumor is essential if we are to develop a treatment that has any hope of containing or ultimately eradicating a brain tumor. To overcome the challenge of intratumoral heterogeneity, a deeper understanding of the cell population structure within a tumor is required to identify the appropriate target(s) against heterogeneous tumor cells throughout the entire tumor.

Taken together, these issues motivate the need for an alternative approach to treat brain tumors, one that can deliver “effective therapy for patients with specific cancer characteristics” [24]. The molecular diversity between tumors, which can appear as phenotypes existing along a continuous range of states or manifest as distinct subtypes of diseases, has motivated the embrace of personalized medicine in oncology. Clinical trials that focus on outcomes related to the biological effect of the drug(s) and evolution of the molecular state of the tumor in response to therapy, in addition to the traditional quantities of progression-free survival (PFS) and overall survival (OS) rates, will provide new dimensions along which to understand the variability of drug efficacy and tumor behaviors, which in turn can translate into the improvement of clinical outcomes. As we continue to explore the depths of tumor complexity, it is clear that a single or even a few approaches are unlikely to provide the comprehensive perspective that is necessary to address the molecular complexity, inter-patient diversity, and intratumoral heterogeneity responsible for the formidable resilience of brain tumors to therapeutic intervention. 

A systems biology approach is designed to address the dynamic, high-dimensional, multi-causal, and multi-scale processes that underlie biological systems [25,26,27]. The integration of HTP (high-dimensional and multi-scale) molecular profiling methodologies with computational capabilities in a cross-disciplinary approach is needed to dissect both inter-patient variability and intratumoral heterogeneity to advance the development of new treatment for brain tumors. A systems approach needs to enable: (1) the stratification of patients into biologically and thus clinically meaningful cohorts, (2) the improvement of our understanding of the biological complexity of brain tumors at the levels of interpatient variability and intratumoral heterogeneity, and (3) the integration of computational data analysis with models to describe and predict the dynamical trajectories of brain tumor progression. The objective is to perform routine high-dimensional molecular profiling and experimental modeling of a patient’s brain tumor to design individualized treatment, based on a new understanding of the principles of tumor progression given the molecular characteristics of each tumor. Such rational, data-driven, patient-tailored approaches would epitomize “precision medicine” in its actual meaning—or “*N*-of-1” medicine [18]. Herein, we briefly review recent technologies, methodologies, concepts, and their applications that would guide or enable a systems approach and pave the path towards realizing this vision. As GBM is the quintessential model of intratumoral heterogeneity, many of the examples discussed focus on this brain tumor type. However, many of the approaches and perspectives presented are applicable across brain tumor types. We conclude by discussing our own example of how several of the methods described herein have been used to characterize an individual tumor and identify plausible drug treatments specific to that patient’s tumor. 

## 2. Molecular Profiling-Based Patient Stratification and Monitoring

**Figure 1:**
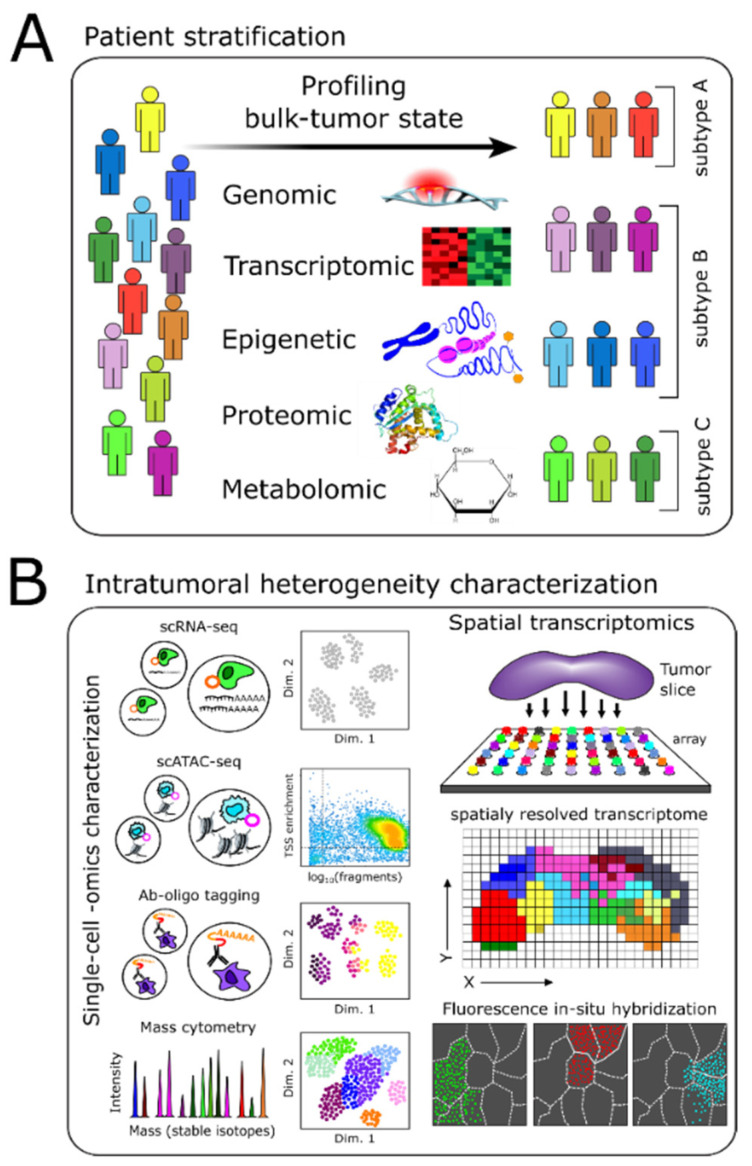
Multi-level characterization of tumors. The convergence of technological advances and existing knowledge of brain tumor biology create an opportunity in which systems approaches can integrate technologies to stratify patients and characterize brain tumors precisely. (**A**) Multi-omic characterization of brain tumors is necessary to stratify patients into biologically and clinically meaningful cohorts that may be more amenable to certain treatments. (**B**) Intratumoral heterogeneity is a major challenge that hinders effective therapy. Multi-omic, single-cell-level characterization on the transcriptomic, epigenetic, and proteomic level is necessary to identify appropriately the various tumor-cell subpopulations and associated molecular vulnerabilities. In addition, spatial transcriptomic technologies provide the ability to measure tumor structure directly, which is lost due to tissue dissociation during sample preparation for single-cell technologies such as scRNA-seq or scATAC-seq.

Until recently, the classification of brain tumors relied largely on the histological characterization of tissue sections based on morphology in hematoxylin and eosin staining and marker protein expression in immunohistochemical staining. Readouts also included the cellular composition of the sample concerning putative cell types and the level of differentiation of the cellular population [28]. Despite being the gold standard for brain-tumor classification, these assays suffer from a lack of reproducibility, significant inter-observer variability, and poor prognostic accuracy. Work over several decades has revealed the extensive genetic, epigenetic, proteomic, and metabolomic heterogeneity of brain tumors, motivating an update to the WHO classification scheme for central nervous system tumors [28]. This new scheme highlights how multiple molecular characterizations enable the better classification of brain tumors to tailor patient therapy, clinical trials, and experimental studies (Figure 1A).

### 2.1. Genome, Epigenome, and Transcriptome Characterization

The greatest improvements in extending the OS of brain tumor patients in recent years have been a result of stratifying patients on the basis of molecular characterization of tumors [24]. Analysis of brain tumors at the cellular and molecular levels has led to the development of an organizational framework within which tumors can be characterized by implications on prognosis and treatment strategies. Technological advances in HTP analysis of tumors at the genomic, transcriptomic, and epigenomic levels have enabled investigators to identify clinically relevant subtypes. Genetic characteristics of brain tumors such as the co-deletion of chromosomes 1p and 19q [29,30,31], mutations in isocitrate dehydrogenase 1/2 (*IDH1/2*) genes [31,32], amplification or mutation of the epidermal growth factor receptor (*EGFR*) gene [33,34], mutations of the promoter region of the telomerase reverse transcriptase (*TERT*) gene [35], and deletion of the phosphatase and tensin homolog (*PTEN*) gene [33,34,35,36] have refined the classification of brain tumor subtypes. Analysis of gene expression and copy-number alterations of brain tumors catalogued in The Cancer Genome Atlas (TCGA) have revealed several distinct molecular subtypes in the case of GBM: mesenchymal, proneural, neural, and classical. These subtype classifications have since been revised, with the removal of the neural subtype deemed to reflect non-tumor cells from the tumor microenvironment [37]. Distinct clinical outcomes associated with these subtypes offer a stratification scheme to achieve more appropriate therapies, albeit not yet at the resolution of the ultimate *N*-of-1 precision medicine. For instance, the proneural subtype is associated with a more favorable outcome, due in part to the high frequency of mutations on the *IDH1/2* genes that lead to a CpG island methylation phenotype (CIMP), which in turn is associated with higher response rates to TMZ than those of other subtypes. By contrast, the mesenchymal subtype is associated with worse survival than that of the other subtypes [37,38,39,40]. 

Genome-wide characterization of “epigenetic marks” has uncovered signatures, such as DNA methylation patterns, that stratify GBM subtypes that are more likely to respond to specific therapies [38,40]. For example, patients whose recurrent tumors exhibit hyper-methylation of the *MGMT* gene promoter region had a six-fold higher PFS in response to TMZ retreatment, demonstrating that molecular marker-based therapies can improve patient outcomes under the right circumstances [41]. Alternatively, analyses of integrated DNA methylation, gene expression, and copy number profiles of samples have revealed the roles of key developmental transcriptional factors in aggressive molecular subtypes with a shorter time to BM [42]. For example, DNA methylation of enhancer elements affecting the cluster of HOXD genes in BM proved to have diagnostic utility in predicting OS in melanoma patients [43]. The combination of genome-scale DNA methylation profiling and copy number variation analysis with machine learning algorithms has also revealed mutually exclusive melanoma BM molecular subtypes corresponding to differences in brain location and outcomes [44,45]. More recently, genome-wide analysis of chromosomal accessibility states of GBM stem-like cells (GSCs) revealed novel subtypes based on sets of distinct transcription factors essential for growth specific to certain subtypes. Targeting these transcription factors would inhibit GSC growth and consequently decrease the risk of recurrence within this particular subtype [46]. Similarly, the mapping of single-cell chromatin accessibility within GBM tumors led to the identification of a novel invasive GSC subpopulation associated with worse clinical outcomes [47]. Ultimately, the characterization of GBM 3D chromatin conformation provided novel therapeutic targets for GSCs. These results highlight how omic-scale characterization efforts have revealed critical mechanisms to tumor growth and improved our ability to stratify patients. 

### 2.2. Proteomics-Based Characterization

While the extension of protein analysis to the omics-scale, or ‘proteomics’, has enabled the discovery and characterization of effectors of disease pathogenesis and treatment response [48], proteomic technologies and their applications to clinical research have advanced to a lesser degree than those of genomics, epigenomics, and transcriptomics. In addition to issues common to both transcriptomic and proteomic assays, e.g., orders-of-magnitude dynamic range in protein abundance and the spatiotemporal complexity of protein expression and interaction, the wide array of post-translational modifications continue to pose challenges towards achieving a comprehensive analysis of the proteome. Despite these challenges, the protein characterization of brain tumors has contributed to multi-factor diagnostic and prognostic signatures that have long been the domain of transcriptomics; certain protein expression patterns can distinguish brain tumors with different clinical outcomes [49] and survival probabilities [50]. 

Mass-spectrometry (MS)-based approaches have accelerated the application of proteomics towards characterizing brain tumors [51,52]. Isotope labeling of proteins has been used to identify protein markers for GBM. Isotope labeling approaches such as stable isotope labeling by amino acids in cell culture (SILAC), tandem mass tag (TMT) labeling, and isobaric tagging for relative and absolute quantification (iTRAQ) have been used to identify protein markers having prognostic value; iTRAQ analysis of sera from GBM patients revealed S100A8/S100A as a predictor of tumor–stroma interactions and prognosis [53]. Alternatively, label-free quantification approaches such as sequential window acquisition of all theoretical spectra–mass spectrometry (SWATH-MS) have enabled the retrospective interrogation of the peptides of interest using spectral libraries and have improved the breadth and depth of measurement of the proteome [54]. Miyauchi et al. used SWATH-MS to analyze blood samples from healthy individuals and GBM patients and identified eight potential biomarker candidates including leucine-rich alpha-2-glycoprotein (LRG1), complement component C9 (C9), C-reactive protein (CRP), alpha-1-antichymotrypsin (SERPINA3), apolipoprotein B-100 (APOB), gelsolin (GSN), Ig alpha-1 chain C region (IGHA1), and apolipoprotein A-IV (APOA4) [55]. 

Beyond biomarker discovery, MS offers great clinical utility in validating, monitoring, and detecting established disease biomarkers. MS-based methods such as selected reaction monitoring (SRM) and multiple reaction monitoring (MRM) measure multiple isoforms and post-translational modifications of proteins in a targeted manner. For instance, changes in several TCA cycle enzymes have been measured via SRM to assess the effect of anti-angiogenic therapy in GBM patients. Decreased levels of isocitrate dehydrogenase and aldehyde dehydrogenase, in response to anti-angiogenic therapy, indicated that tumor cells could evade death by increasing glycolysis [56]. Alternatively, protein arrays and antibody-based protein quantification offer highly sensitive quantification methods for clinically testing and monitoring protein biomarkers. Recent advances in multiplexing capabilities at the single-cell level have been achieved using nucleotide barcode sequence-labeled antibodies that enable tag amplification and consequently signal amplification [52,57]. When integrated with transcriptomics and gene editing, such technologies offer the potential to perform multi-modal characterization of the heterogeneous tumor cell population [58]. Most recently, concerted efforts by Wang et al. characterizing the proteogenomic and metabolomic landscape of 99 GBMs revealed key phosphorylation events (e.g., phosphorylated PTPN11 and PLCG1) as potential switches that mediate oncogenic pathway activation. The in-depth proteomic characterization offers a valuable resource for future GBM studies [59].

### 2.3. Metabolomics-Based Stratification and Monitoring

Altered metabolism is a clinically actionable hallmark of cancer [60]. A primary metabolic feature of cancer cells is their preference to produce energy via glycolysis, even in the presence of oxygen, rather than the more efficient process of mitochondrial oxidative phosphorylation, which normal cells utilize for energy production. Known as the Warburg effect [61], this metabolic shift may support the high energetic demand for the uncontrolled proliferation of cancer cells. While the precise function of the Warburg effect remains unclear [62], there is mounting evidence that dysregulation of the associated metabolic enzymes and pathways, assessed by genomic, epigenomic, transcriptomic, and metabolomic measurements, may have enormous utility as theranostic markers. In GBM, gain-of-function mutations in the *IDH1* gene lead to increased production of oncometabolites such as 2-hydroxyglutaric acid (2-HG), which inhibit histone demethylases and the TET family of 5-methycytosine (5mC) hydroxylases. Studies have indicated that the inhibition of 2-HG induces the demethylation of histone H3K9me3, which promotes glioma-genic differentiation and reduces tumor cell viability in vitro and in vivo [63]. 

Improvements in MS technologies for proteomic analyses have also translated to metabolite analysis. Gas chromatography (GC)- and liquid chromatography (LC)-MS have enabled in-depth metabolic profiling of brain tumor samples [59]. GC-MS has been used to measure metabolic profiles in cerebrospinal fluid across primary brain tumors (grades I to IV). Although no differences in metabolic profiles were found, trends between elevated levels of citric and lactic acid and lower OS were observed across all samples, suggesting potential prognostic capabilities [64]. 

Alternatively, noninvasive metabolically based imaging techniques have resulted in several insights into the altered metabolism of tumor cells. For example, magnetic resonance spectroscopy (MRS) combined with isotope labeling, which measures radiofrequency signals generated by the nuclear spins of magnetic-resonance-active nuclei including hydrogen-1 (^1^H), phosphorus-31 (^31^P), nitrogen-15 (^15^N) and carbon-13 (^13^C), has been used to detect metabolic routes and fluxes in both tumor and non-malignant tissues in vivo [65]. ^13^C MRS studies in GBM revealed that the majority of acetyl-CoA, a central metabolite for energy production and biosynthesis, was not derived from glucose, suggesting that non-glucose carbon sources may be contributing to bioenergetic processes in GBM. Similarly, ^13^C tracing studies have shown acetate to be a critical substrate from the microenvironment, contributing to bioenergetic processes in both GBM and brain metastases [66]. 

Positron emission tomography (PET) measures positron-emitting radioactive isotopes including ^11^C, ^13^N, ^15^O, and ^18^F from an injected tracer. Placing these tracers onto a metabolite of interest allows one to evaluate the uptake and retention of the probe, which is anatomically registered using computed tomography or magnetic resonance imaging (MRI). PET, therefore, reports metabolic activities rather than static metabolite levels. A commonly used radioisotope is ^18^F-labeled glucose-analogue tracer 2-[^18^F]fluoro-2-deoxy-d-glucose (^18^F-FDG), which cannot be metabolized and thus accumulates in cells [66]. Based on the high glucose demand of brain tumor cells, ^18^FDG-PET is useful for cancer diagnosis, staging, and monitoring drug treatment efficacy—the idea being that the therapeutic response is accompanied by a reduced PET signal that can be detected long before reductions in tumor size [67]. Such non-invasive measuring tools provide highly relevant diagnostic/prognostic capabilities, as well as the ability to monitor the effects of drugs targeting metabolic dependencies [68], all of which are essential in stratifying and monitoring patients with brain tumors.

Concomitantly, rapid developments in analytical tools have made once complex metabolic flux analyses (MFA) and metabolic network mapping more accessible to non-experts. In particular, ^13^C-MFA is a popular technique for quantifying intracellular flux in tumor cells [69]. Although widespread use of ^13^C-MFA has not yet been established amongst cancer biologists, this method offers a powerful approach to identify differentially active metabolic pathways and promises to facilitate the identification of metabolic dependencies or vulnerabilities in tumor cells. Such methods will provide novel insights into metabolic differences across heterogeneous tumor cell populations and compliment current imaging methods, enabling the clinical translation of yet-to-be discovered insights. 

### 2.4. Liquid Biopsies and Longitudinal Monitoring of Patients

In addition to patient stratification, an important question to consider in clinical care is how drug treatments affect the molecular state of the tumor and its microenvironment. To address these questions, highly sensitive, specific, and repeated measurements are required to longitudinally monitor the molecular responses of patients and assess treatment efficacy. Towards this end, liquid biopsies are gaining traction as a surrogate for tumor biopsies as a means to assess and manage primary and metastatic brain tumors. The practical significance of a liquid biopsy is two-fold. First, the very presence of material derived from the tumor per se would have diagnostic and prognostic significance [64]. Second, the analysis of circulating tumor-derived material can inform on multiple aspects of the tumor and provide insights into the effect(s) of drug/treatment and facilitate the early detection of tumor recurrence [70].

Briefly, liquid biopsies detect and analyze circulating tumor cells (CTCs), circulating tumor DNA (ctDNA), cell-free nucleic acids (cfNA, e.g., DNA, RNA, or miRNA), and exosomes that are present in bodily fluids, primarily plasma, from which multiple genetic and epigenetic alterations and relevant tumor biomarkers can be assessed [71]. Analytical platforms for liquid biopsies rely on PCR or next-generation sequencing (NGS) technologies. PCR-based techniques such as digital PCR (dPCR) effectively perform single-locus or loci-multiplexed assays of a priori determined targets. For example, mutant allele fractions (MAF) of less than 0.1% have been reported with dPCR [72]. Similarly, the EGFRvIII variant has been observed across glioma cell lines, xenograft mouse models, and patient-derived tumor specimens at a detection rate of 0.003% [73]. Alternatively, NGS-based techniques enable unbiased genome-wide sequencing but vary in their sensitivity and specificity in measuring genomic abnormalities. Lower detection limits have been reported to be as low as 0.1% to as high as 10% [70]. 

While ctDNA correlates with tumor burden and progression in colorectal, breast, lung, and ovarian cancer patients [64], this correlation does not hold in brain tumor patients. Often, plasma ctDNA in those patients is present in amounts lower than what is observed in other cancer-type patients, or not at all [74]. However, cerebral spinal fluid (CSF) has been used as a viable source to measure ctDNA and related tumor characteristics [75]. Actionable mutations in multiple genes (e.g., *EGFR, PTEN, ESR1, IDH1, ERBB2,* and *FGFR2*) and longitudinal changes in MAFs following surgical treatment have been detected in cfNA present in CSF of GBM and BM patients [74]. Similarly, clinically actionable mutations in CSF-associated ctDNA have been measured in a large but varying percentage (>50 to 100%) of brain tumor patients across multiple studies [76,77,78].

These examples support the clinical validity of such assays, i.e., accurate detection of the presence/absence of a pathologic state or outcome. However, clinical validity is not a guarantee of clinical utility, i.e., the demonstration that ctDNA-informed treatment improves prognosis over treatment without it [79]. The most reliable method to establish clinical utility is a prospective clinical trial. One such trial suggests that cfNA in pre-operative plasma is an effective surrogate for GBM tumor burden and the detection of somatic alterations [80]. Although the absence of any detectable CTCs, ctDNA/cfNA is far from indicative of the absence of a tumor, their positive detection and analysis of their features can offer a glimpse of the molecular nature of the brain tumor. Continued mapping of the molecular landscape of brain tumors will eventually translate key tumor biomarkers into targeted liquid biopsy assays. In the grander vision, blood biopsies may offer a “looking glass” to tumor progression or resistance to treatment, thus informing *N*-of-1 therapy.

## 3. Intratumoral Heterogeneity 

The prevalence of phenotypic heterogeneity of cells within a tumor has been observed since the earliest days of cancer biology [81]. The pervasiveness of phenotypic heterogeneity across cells within a tumor negates the effectiveness of a single-target therapy. The presence of small, rare subpopulations of inherently chemotherapeutic-resistant cells, i.e., stem-like cancer cells [82] or tumor cells predisposed to acquire resistance upon drug treatment [83], further complicates matters. Overcoming intratumoral heterogeneity requires advanced technologies to characterize tumors at the necessary level of precision, i.e., the single cell, and a deeper understanding of the factors underlying heterogeneous cell population structure within a tumor (Figure 1B).

### 3.1. Methods Enabling Single-Cell Level Characterization of Tumors

HTP single-cell sequencing technologies, primarily scRNA-seq, have enabled incredibly precise investigation into cell–cell heterogeneity. SMART-seq generates full-length cDNA, providing read coverage across the entire transcript, enabling the detection of single-nucleotide polymorphisms (SNPs). Alternative protocols such as massively parallel scRNA-seq (MARS-seq), single-cell tagged reverse transcription (STRT), cell expression by linear amplification and sequencing (CEL-seq), and DROP-seq incorporate unique molecular identifiers (UMIs) into partial-length cDNAs. When coupled with microfluidic or droplet-based platforms, these protocols facilitate HTP sample preparation and have been extensively reviewed [84,85,86,87]. An alternative to measuring transcriptional heterogeneity in the brain is single-nucleus RNA sequencing (snRNA-seq) [88], a method that measures the transcriptome within the cell nucleus and has shown concordance with single-cell transcriptomic profiles [89,90,91]. Two main advantages lie in snRNA-seq: first, nuclei, as compared to the whole cell, are easier to isolate from complex tissues such as the brain. Easier nuclear isolation avoids tissue dissociation steps that can induce a stress response in cells and affect the final transcriptomic output. Second, snRNA-seq produces high-quality transcriptomic profiles from frozen samples. This is highly beneficial when analyzing human brain samples for which often only frozen samples are available. The use of snRNA-seq will continue to increase, particularly as toolboxes that aid in selecting an appropriate transcriptomic assay emerge to help inexperienced users [92]. One caveat to using snRNA-seq, however, should be noted—a recent report showed that snRNA-seq does not detect the expression of certain genes associated with microglial activation [93]. Given the complex yet influential roles that microglia/macrophages play in brain tumor maintenance, growth, and drug resistance (Section 3.4. Tumor Microenvironment), this gap should be considered when selecting a transcriptomic profiling method for analyzing a brain tumor. 

Beyond the transcriptome, recent technological advances such as single-cell assay for transposase-accessible chromatin sequencing (scATAC-seq) have enabled epigenetic characterization of single cells, which provides an orthogonal analysis of regulatory mechanisms associated with cell–cell heterogeneity [94,95]. Furthermore, the integration of these methods is beginning to provide multi-modal characterization of single cells. One example includes the coupling of antibody-based detection with scRNA-seq, which enables the measurement of select cell-surface proteins and transcriptome within individual cells [96]. Now, protocols have been developed that integrate scRNA-seq and scATAC-seq, which enable the simultaneous measurement of the transcriptomic- and chromatin accessibility-state of individual cells (10x Genomics, Pleasanton, CA, USA). 

The spatial architecture of a solid tumor is another important aspect that can shed light onto cell–cell interactions such as those occurring between the leading tumor edge and tumor microenvironment, which cannot be discerned easily from scRNA-seq. Fluorescence in situ hybridization (FISH), for example, provides spatially resolved transcriptional patterns [97]. Although omics-scale analyses are not yet possible with FISH, multi-transcript profiling in a single sample via FISH has been achieved up to a panel of 19 marker genes [83]. Alternatively, Slide-seq produces spatially resolved RNA-seq profiles within tissue sections [98]. Originally, Rodriques et al. developed Slide-seq to discern the temporal evolution of cell type-specific responses to traumatic brain injury in a mouse model. In a related manner, Liu et al. developed the deterministic barcoding in tissue for spatial omics sequencing (DBiT-seq) [99]. Here, microfluidic-based technologies are used to apply oligo-tagged DNA barcodes directly on the surface of tissue slices in a 2-D fashion. The result is a 2-D coordinate system of pixels, where each pixel contains a unique combination of oligo-tagged DNA barcodes. The tissue is then digested to recover spatially barcoded cDNAs, which are then template-switched, PCR-amplified, and tagmented for library preparation for NGS sequencing. Proteins can also be co-measured by applying a cocktail of antibody-derived DNA tags (ADTs) to the fixed tissue slide prior to flow barcoding, similar to Ab-seq [57] or CITE-seq [96]. Similar technologies are now becoming more widespread through commercialization efforts (e.g., GeoMX^TM^, NanoString^®^ Seattle, WA, USA; Visium, 10x Genomics), which enable multi-modal analysis of FFPE sections. Together, single-cell and spatially resolved profiling will elucidate the spatiotemporal dynamics of cellular interactions that enable the survival and uncontrolled proliferation of heterogeneous tumor cell populations. 

### 3.2. Characterizing Genetic and Non-Genetic Cell–Cell Heterogeneity

Single-cell (phenotypic) heterogeneity results from a combination of genetic and non-genetic factors. The prevailing paradigm of clonal diversity arising from Darwinian-like evolution of a single cell gaining a random oncogenic mutation offers a plausible yet incomplete view of tumor cell heterogeneity. In this gene-centric framework, clinically relevant phenotypic heterogeneity can be explained by the genetic diversity that arises from clonal evolution [81,100]. However, research aligned with cancer stem cell (CSC) theory challenges this gene-centric perspective. Under the CSC hypothesis, the functional diversity of tumor cells arises from differences in differentiation states that exist within a (dysfunctional) developmental hierarchy, rather than solely due to the accumulation of genetic mutations. More generally, the inherently stochastic and non-linear nature of biological processes, such as gene locus activation and gene expression, can drive cells with the same genome into phenotypically distinct, stable states that can be inherited across cell generations [21,22,101,102]. Below, we discuss a few examples highlighting both genetic and non-genetic mechanisms that contribute to intratumoral heterogeneity and inform our approach to treating brain tumors.

#### 3.2.1. Genetic Intratumoral Heterogeneity 

Certainly, genomic instability can result in somatic mutations and provide an avenue for evolutionary selection, a powerful mechanism that can lead to the emergence of drug-resistant tumor cells. Multiple efforts have demonstrated subclonal diversity within a tumor, in which subclonal populations derived from single-cell clones isolated from a tumor display distinct phenotypic properties including growth rates, biomarker expression, mutational profiles, and drug sensitivity [103,104]. Indeed, some preexisting subclonal populations in treatment-naïve GBMs are resistant to TMZ [103]. Moreover, a single GBM tumor can contain subpopulations of cells corresponding to different GBM molecular subtypes as well as individual tumor cells that exhibit transcriptional profiles similar to multiple subtypes [23]. At the genomic level, mosaic amplification of key oncogenes in mutually exclusive regions of a tumor can functionally distinguish subpopulations [105,106]. For example, mosaic amplification of *EGFR* and *PDGFRA* genes in GBM cell lines required simultaneous inhibition to achieve pathway inhibition [107]. Recent efforts characterizing single-cell heterogeneity across 28 GBM tumors defined dominant cell states, which tumor cells can transition into and out of, with certain states being favored by cells having certain copy number aberrations (e.g., amplifications of *CDK4*, *EGFR*, and *PDGFRA* loci) [108]. These results provide a multi-level framework within which we can interpret the genetic programs contributing to GBM tumor cell heterogeneity. 

#### 3.2.2. Non-Genetic Intratumoral Heterogeneity

Concomitantly, it is less appreciated that non-genetic mechanisms such as epigenetic modifications, differentiation gene-expression programs, and stochastic gene expression can establish and maintain distinct functional states as well as drive individual clonal cells into these states in a stochastic or regulated manner [109]. Shaffer et al. confirmed the old idea that transcriptional variability within an isogenic cell population supports the presence of rare cell types that differ not only in their drug sensitivity but also in their propensity to undergo transcriptional reprogramming, resulting in drug-induced resistance [83]—a mode of treatment-resistance development that fundamentally differs from the selection of preexisting resistant clones and operates at much faster time scales. Similarly, Dirkse et al. demonstrated that environmental interactions and conditions such as hypoxia triggered phenotypic plasticity of multiple GBM tumor cell populations [110]. This plasticity enabled tumor cells to undergo reversible transitions into a stem-like cell state, independent of clonal population structure. Likewise, transitions into a stem-like cell state could be triggered by manipulating defined transcriptional factors in vitro [111], further supporting the idea of non-genetic mechanisms underlying phenotypic heterogeneity across tumor cells. 

Compelling evidence generated by Lan et al. [112] suggests that the heterogeneity in clonal expansion observed in GBM results from normal developmental processes that generate the hierarchy of cell types rooted in GSCs, independent of an evolving mutational signature. They also showed that distinct GSC populations, such as pre-existing drug-resistant GSCs, can be targeted by epigenetic compounds [112], emphasizing the importance of non-genetic sources of intratumoral heterogeneity and their value as targets for treatment. Finally, distinct metabolic demands of GSCs offer another distinguishing feature. GSCs consume less glucose than tumor cells and would require distinct targeted treatments in order to be eradicated and prevent tumor recurrence [113]. Given the multiple avenues through which intratumoral heterogeneity may arise, multidimensional phenotyping of tumors will greatly inform treatment design. 

### 3.3. Spatial Heterogeneity

Genetic and non-genetic heterogeneity manifests non-uniformly throughout the tumor space. This spatial heterogeneity results in an underestimation of these aberrations otherwise portrayed by a single tumor-biopsy sample [114]. Spatial heterogeneity is a product of multiple intrinsic and extrinsic factors and their reciprocal interactions. As these genetic and non-genetic differences spread throughout the tumor space, they manifest themselves through different modes. 

Multiple forces influence genetic heterogeneity observed throughout the tumor structure. Selective sweeps, genetic drift, selection, and clonal dynamics, which itself is dependent upon rates of clonal expansion and mutation, all affect how genetic variations affect the spatial distribution of clonal populations throughout the tumor. Under the traditional clonal evolution model, a sequential acquisition of mutations would occur with concomitant, successive clonal dominance or selective sweeps throughout the tumor, which is supported by histopathology of disease progression in adenoma, carcinoma, and metastases [115]. However, multiple findings present a more complex picture of the evolutionary trajectories occurring within a tumor. Analysis of SNPs in multiple types of childhood cancers (neuroblastoma, Wilms tumor, and rhabdomyosarcoma) suggests that a finite number of evolutionary trajectories underlie intratumoral genetic variation, resulting in distinct clonal architectures in solid tumors [116]. Karlsson et al. analyzed 250 regions across 54 tumors and identified four distinct trajectories: (1) clonal sweep, where an emergence of a clone with driver mutation(s) sweep an anatomical region; (2) subclonal variation, where subclones were confined to a single region; (3) the stable coexistence of clones, where multiple clonal populations spread throughout the tumor space; and (4) the emergence of a myriad of clones, i.e., regional evolutionary explosion [116]. In addition, the type of evolutionary trajectory taken by a clonal population is subject to the reciprocal interaction between tumor cells and their microenvironment, where regional differences of resources can affect clonal evolution rates [117] or result in cell migration and emigration [115]. 

In the context of GBM, the Ivy Glioblastoma Atlas Project [118] mapped transcriptional and genomic differences across four distinct regions in each of the GBM tumors analyzed across a cohort of 41 patients, which further highlighted spatial heterogeneity previously reported in GBM tumors [105,119]. A subsequent study involving the multiregional sampling and genomic profiling of 15 distinct tumor fragments from seven different GBM tumors revealed a similar inconsistency in the distribution of mutations across tumor fragments. In *IDH1*-mutant tumors, *IDH1* and *FGFR3-TACC3* mutations were present across all corresponding fragments. In contrast, *PTEN* alterations were only observed in a subset of tumor fragments. Similarly, *EGFR* amplifications were found to be exclusive to only one or two regions of the multiregional samples, suggesting that genetic information obtained from a single biopsy may not be representative of the entire tumor [120]. Taken together, it is apparent that the application of NGS at the bulk and single-cell levels applied to multiregional samples (when feasible) is critical in identifying biomarker(s) and target(s) that would inform the treatment strategy for an individual patient. 

As non-genetic mechanisms can act as the interface between the environment and the genome, structural niches directly contribute to the non-genetic, spatial heterogeneity observed in brain tumors. Distinct microenvironments can alter signaling patterns, gene expression, and other non-genetic mechanisms that can lead to heritable phenotypic differences. Distinct hypoxic and normoxic regions within a tumor [110], spatial variation of cell-type proportions, and the tumor microenvironment contribute to differences in cell states and phenotypic behavior [121], manifesting as decreased tumor sensitivity to therapy [122]. 

### 3.4. Tumor Microenvironment (TME)

The reciprocal interactions occurring among tumor cells and the multiple cell-types therein also affect the population structure. Interactions among malignant cells, infiltrating immune cells, neovascularization, and surrounding non-malignant cells in the TME help shape the brain tumor phenotype [123]. Single-cell-level investigation into the microenvironment in metastatic melanoma revealed that the number of tumor cells exhibiting drug-resistant transcriptomic states correlated with the proportion of endothelial cells in the surrounding microenvironment, suggesting that interactions between tumor cells and the microenvironment likely shape the tumor cell transcriptome [121]. 

Concomitantly, cell–cell interactions, inferred from single-cell analyses of tumors, have provided another quantifiable metric with which to characterize tumors and inform target identification [124,125]. Kumar et al. identified a strong correlation between tumor growth and interactions involving the extracellular matrix (ECM) and integrin receptors, which corroborates the known role of the ECM in modulating tumor growth. Importantly, their analysis revealed that several interactions, when viewed in different cell-type pairs, had opposite correlations with a particular tumor phenotype, supporting the pleiotropic role that cell–cell interactions play. Consequently, careful examination of the multiple effects that interactions have on different cell populations should be made in order to understand the broader impact of targeting a particular interaction [125]. One can imagine how similar insights gained from this type of analysis of the microenvironment would inform efforts to identify novel targets to treat brain tumors. 

Given the influential role that the TME plays in brain tumor progression, significant efforts have been made into characterizing the TME and its constituent components. Comprised of neoplastic and non-neoplastic cells, a major class of cells that constitute the TME are tumor-associated macrophages (TAMs), which play a critical role in tumor maintenance, invasiveness, and drug-resistance [126,127]. Briefly, TAMs comprise a mixed population of resident microglia and peripheral macrophages, making up approximately 40% of the tumor mass [128,129]. Depending on the signals received by the recruited macrophages, these cells can polarize along a continuum of states, often described by their two extremes, the M1-state (anti-tumor, pro-inflammatory phenotype) and M2-state (pro-tumor, anti-inflammatory, phenotype). Anti-tumor properties of M1-like TAMs include tumor-cell killing abilities, which are mediated by the production of NO, ROS, and IFNγ [130,131]. M1-like TAMs mediate the Th1 response in the tumor through the activation of Th1 helper cells. Conversely, M2-like TAMs exhibit a variety of pro-tumor properties. M2-like TAMs express cytokines such as IL-6, IL-10, and TGFβ1, which activate signaling pathways promoting tumor-cell proliferation and invasion. Interactions between M2-like TAMs and endothelial cells can lead to angiogenesis through the expression of TGFβ1 and integrin αvβ3, which induces SRC-PI3K-YAP signaling [126]. In addition, TGFβ1 expression can result in the recruitment of CD133+ cancer stem-like cells. Furthermore, M2-like TAMs promote immunosuppression though the expression of IL-4Rα, IL-6, MIC-1, MIF, STAT3, and TGFβ [126]. 

While genetically stable, TAMs can alter their gene expression profiles depending on the signals they receive or interactions in which they are involved. Thus, interactions with heterogeneous tumor-cell subpopulations will result in heterogeneous TAMs. Analysis of TAMs via scRNA-seq indicated that they can express signatures for both M1 and M2 states [127]. Similarly, TAMs exhibit extensive functional diversity. Ochocka et al. recently showed in murine models that glioma cells attract and polarize microglia and monocytes [132]. While these cells retained their cell-type signatures, they occupied different tumor niches and displayed varying degrees of tumor-induced activation. Interestingly, investigation into sex-related differences of TAMs revealed distinct microglial responses to glioma where male monocytes and macrophages showed higher expression of MHCII-related genes, which could have implications in targeting TAMs across sexes [132]. Moreover, single-cell profiling of TAMs in human GBM and murine models revealed that microglia- and monocyte-derived TAMs are dynamic populations wherein microglia-derived TAMs were dominant in newly diagnosed tumors, whereas monocyte-derived TAMs were dominant in recurrent tumors, particularly in hypoxic regions of the tumor [133]. As resident microglia are involved in several pro-tumor processes such as the evasion of antitumor immune response through the down regulation of major histocompatibility complex class II (MHC II) via TLR2 expression [134], these results provide insight into which mechanisms are at play in primary and recurrent tumors. Given the substantial role that TAMs play in tumor progression, several strategies are being developed to target TAMs by blocking TAM recruitment, or reprogramming TAMs into an anti-tumor phenotype [126,127]. While much remains unclear about TAMs, understanding their role in brain tumor progression will continue to play a critical role in improving treatment.

## 4. Vertical Data Integration and Computational Analyses

**Figure 2:**
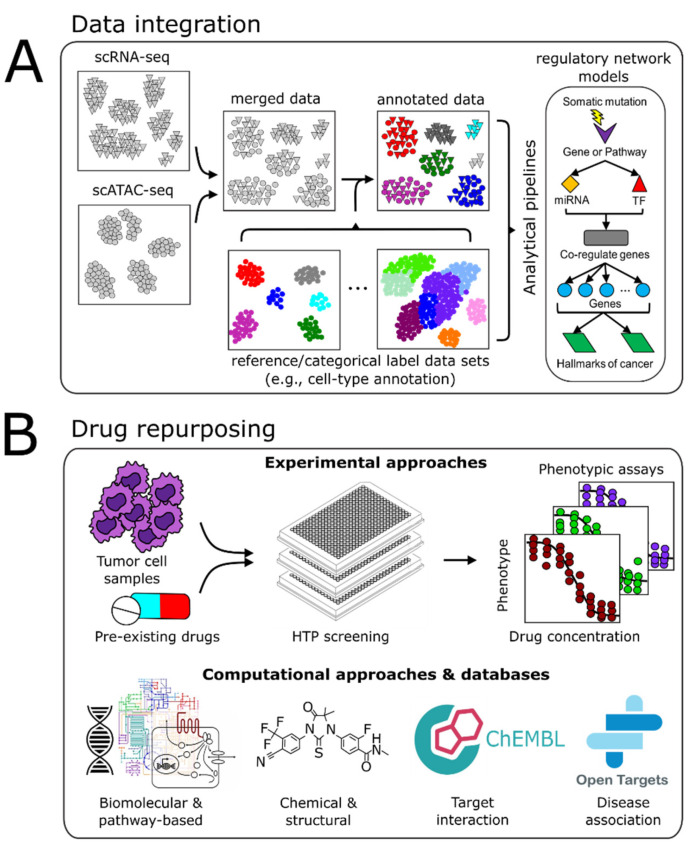
Data integration and drug repurposing. (**A**) Multiple computational approaches have been developed to accomplish a variety of complex tasks such as integration of datasets from the same modality, e.g., scRNA-seq; or from different modalities, e.g., scRNA-seq, scATAC-seq. Using reference datasets or established cell-type marker gene sets, integrated datasets can then be annotated accordingly. These results can be used as inputs to computational pipelines to infer transcriptional regulatory networks underlying observed single-cell phenotypes throughout the tumor. This analysis can lead to the identification of novel molecular targets. (**B**) Current drug repurposing approaches involve both experimental and computational approaches. Experimentally, high-throughput (HTP) screening methods enable drug panels to be screened against model cell lines or patient-derived samples efficiently. Computationally, the development and continual curation of databases provide a plethora of information enabling one to match pre-existing drugs to novel molecular targets that have the potential to target various tumor cell subpopulations having distinct phenotypes, i.e., molecular vulnerabilities.

As the size of datasets and the rate of data generation continue to increase, so too does the need for bioinformatics tools, computational algorithms, and comprehensive models that enable efficient analysis, integration, and interpretation of these multi-omic, multi-scale datasets to relate genotype to phenotype. This “vertical” data integration (genotype to phenotype) requires a systems perspective to infer predictive statistical models that facilitate the discovery of novel biological insights and advance clinical applications. However, a major challenge is managing the breadth and depth of data generated and how these multi-modal data-types from different biological levels (transcriptional, post-transcriptional, epigenetic, metabolomics, etc.) can be integrated appropriately. Multi-scale data integration and analyses are necessary to uncover causal and mechanistic flows of information that can explain and predict how specific molecular changes modulate complex phenotypes (Figure 2A).

### 4.1. Batch Integration 

Along with the continual development of aggregate algorithms and computational pipelines, the amount of single-cell, multi-omic data continually increases. To maximize insights from multi-omic data derived from individual patients or cohorts, the ability to integrate disparate datasets within and across technological platforms and data modalities is essential. While data normalization addresses differences in expression distribution across samples within a batch, technical differences in sample processing across batches remain. Towards addressing these issues, several methods have been developed to account for batch effects across microarray and bulk RNA-seq datasets [135,136,137,138,139,140]. The success of these methods, however, depends on certain assumptions of conserved gene expression structure and uniformly distributed batch effects, which do not necessarily apply to single-cell data, where various cell types or cell states are represented unevenly in a single-cell sample population. Consequently, multiple single cell-specific methods have been developed to address these single cell-specific issues, which have been reviewed and whose performances have been benchmarked elsewhere [141,142]. 

Briefly, a majority of these methods involve some form of batch-specific normalization, scaling, and feature, i.e., gene selection, which involves removing all but the most variable genes (e.g., top 5000) to create a gene set large enough to identify rare cell populations. The consideration of these highly variable genes across datasets forms the basis for integration. Next, the integration process involves the identification of a lower-dimensional space common to all samples and datasets. An underlying assumption is that cells of the same type or cells in the same state will map closely to one another when projected into the lower-dimensional space, even if cells originated from different experimental conditions. In this lower-dimensional space, distances among cells are then used to align cells across batches. A prerequisite for this approach is that there exists at least one common cell population across the batches being integrated. Distances among the projected cells form the basis of correction vectors, which rely on a select group of reference cells (anchors), and guide the alignment of the remaining cells across batches. Alternatively, the distances in the low-dimensional space can be used to create a joint-graph representation of the data, from which a variety of graphical-based methods can be applied to identify highly interconnected communities in the graph network and align batches [141]. 

Correction vector or anchor-based alignment methods employ a variety of supervised or unsupervised approaches to define anchoring cell pairs. A general class of methods relies on mutual nearest neighbors (MNN) to identify pairs of cells that are closest to each other across all single cells. Examples of these approaches include Scanorama [143], BEER [144], and Seurat v3 [145]. Joint-graph methods that rely on identifying anchors within a graphical network representation of the data include CONOS [146], BBKNN [147], and scPopCorn [148]. A distinguishing feature between anchor-based and graph-based approaches is how edges that connect single-cell samples within or across batches are defined. These methods are just a few examples of the various methods currently available (https://scrna-tools.org accessed on 22 July 2021). 

Finally, an important step to batch integration is cell-type annotation, which frames the tumor cellular landscape and facilitates tumor subtype classification. Multiple techniques are available to merge and annotate single-cell data based on a priori data or annotate cells de novo [141]. As more scRNA-seq datasets become available, their accurate integration into pre-existing databases will increase the robustness of scRNA-seq signatures of distinct cell-types. The increasing robustness of these reference signatures will improve our ability to characterize cell population structures and identify specific subpopulations that can be targeted therapeutically. 

### 4.2. Multimodal Data Integration

Beyond technologies that simultaneously profile specific surface proteins and transcriptome within a single-cell [57,96], orthogonal omic-scale single-cell technologies such as scRNA-seq and scATAC-seq promise to yield tremendous insights into the cell states and regulatory mechanisms driving tumor pathology. Simultaneous multi-omic single-cell profiling capabilities are currently available and have been reviewed previously [84,149,150]. Conversely, multimodal characterization often occurs in different sample cell populations from the same tumor. In this case, computational methods similar to those described in the previous section are required to integrate and analyze multi-modal data. Here, data integration techniques rely on the underlying assumption that cells of the same type exist in the same state share set(s) of correlated features. These shared features then drive the combination and matching of signals across orthogonal datatypes. For instance, scATAC-seq data can be linearly transformed into gene expression correlates, where they are then integrated with scRNA-seq data [145,151].

Multiple examples demonstrate how multimodal profiling of brain tumors has provided insights into tumor progression. Recently, Dekker et al. performed a transcriptomic, proteomic, and phosphoproteomic analysis of matched primary and recurrent tumors across eight patients [152]. Interestingly, proteomic rather than transcriptomic analysis showed differences between the matched recurrent and primary tumors analyzed across eight patients. Subsequent integration of the data modalities with signaling and pathway revealed that each patient had distinct sets of signaling pathways that were either up- or down-regulated including neuroinflammation signaling, LXR/RXR activation, and atherosclerosis signaling, further highlighting the challenge of selecting treatments due to inter-patient variability. Wang et al. used a combination of scRNA-seq, scATAC-seq, and exome-seq to characterize a cohort of *IDH*-wild-type GBM tumors [153]. Their multi-modal analysis revealed that GBM tumors contained hierarchies of mesenchymal and proneural GSCs and their corresponding differentiated progeny. The combination of variable GSCs, their progeny, and stromal/immune cells was sufficient to explain the transcriptional heterogeneity underlying GBM. Their use of scATAC-seq also revealed cell type-specific cis-regulatory sets of rules, or grammars, associated with distinct neoplastic cell states. This characterization led them to identify synergistic drug combinations that targeted both the proneural and mesenchymal GSC phenotypes present. These results provide an intriguing example of how multi-modal and multi-level characterization can yield tremendous insights into intratumoral heterogeneity and inform drug selection/treatment. 

### 4.3. Computational Pipelines

The development of computational workflows or “pipelines” that aid investigators in compiling, formatting, and analyzing large, multi-modal datasets has been beneficial in dealing with large omics datasets. Such pipelines can be designed to facilitate the integration of multiple data modalities to generate models that take into account the genome-wide regulatory machinery that governs cell states. One such example, SYGNAL [154], integrates omics-scale data analysis techniques with mechanistic principles of gene regulation and a priori knowledge of regulatory interactions. The development of a SYGNAL-based transcriptional regulatory network model from the analysis of TCGA GBM samples revealed a complex set of disease-relevant modules that led to the identification of synergistic drugs [154], providing an example of how a systems biology perspective offers a rational approach to identifying useful drug combinations. Other network-based computational approaches rely on techniques from graph theory; identifying groups of highly interconnected nodes, which can represent genes, proteins, and other key biological components, or modules that underlie key cellular processes may have practical utility. The identification of frequently perturbed modules in tumor samples could help to eventually replace the outdated concept of ‘driver’ mutations and instead guide the targeting of entire causative pathways. This would obviate the need for categorizing mutations as ‘driver’ or ‘passengers’ merely based on their frequent occurrence in associated tumor types because mutations may be unique to individuals. It is in this sense that network analysis can be used to identify the potential functional impact of specific gene mutations [155]. 

Given this perspective, a network-based approach can be used to verify, refine, and build upon prior predictions generated from the use of methodologies such as MutSigCV [156] and MuSIC [157], focused on distinguishing so-called ‘driver’ mutations or pathways from ‘passenger’ mutations. The same refinement of predictions from bioinformatics and machine learning-based methods that infer the functional impact(s) of non-synonymous amino acid changes by analyzing conservation of amino-acid sequences [158,159] can be incorporated into network-based analyses. This can also be extended to the level of signaling pathways where methods such as gene set enrichment analysis (GSEA) [160], which aims to identify which pathways may be over/under-represented from differentially expressed genes, may help assign potential functional impacts of non-recurring mutations. 

## 5. Drug Repurposing

Formulating combinations of drugs that target multiple vulnerabilities identified within or across tumor-cell populations is essential to realize the *N*-of-1 precision medicine paradigm. The FDA has approved over 20,000 prescription drugs for a myriad of indications [161]. Currently, there are 628 approved cancer drugs [162]. In 2020, 18 cancer therapies were approved, making up a dominant 34% of all approved drugs that year [163]. While the continued focus remains on approving novel cancer drugs, a new paradigm has emerged that aims to repurpose existing FDA-approved drugs to treat indications outside of their original purpose. Prior examples of drug repurposing have been serendipitous in nature [164]. By contrast, a systems biology approach can be used to systematically uncover unique vulnerabilities within a tumor by revealing tumor-cell- and transcriptional-network characteristics that could potentially be targeted or inform on drug-responsiveness in patients. Such information would facilitate the design of clinical trials with precise recruitment criteria, which would result in the assembly of a more appropriate patient cohort. In turn, this would improve the probability of detecting statistically significant effects for patient-matched therapies and lead to a greater number of opportunities to repurpose approved drugs for a personalized treatment regimen (Figure 2B). 

### 5.1. Experimental Approaches

Molecular binding studies seek to identify binding partners for drug candidates and involve proteomic techniques such as affinity chromatography and MS. Knowledge of drug binding targets and off-target binding is critical to drug repurposing. HTP direct binding or catalytic assays, where small-molecular-kinase binding is analyzed, have been used to detect novel drug–target interactions [165]. Similarly, phenotypic screening aims to identify compounds that show disease-relevant effects in model systems such as established cancer cell lines or patient-derived tumor cell cultures in vitro. Typically performed in an HTP manner, a panel of approved drugs and/or experimental compounds are screened on a model system of interest using viability or proliferation measures, for example, as a primary endpoint. Disulfiram, an aldehyde dehydrogenase (ALDH) inhibitor used for alcohol abuse, has been identified as a compound that could inhibit proliferation in both prostate cancer cell lines [166] and patient-derived GBM stem cells [167]. Consequently, disulfiram is being tested on GBM patients in clinical trials (NCT01907165, NCT03363659). 

### 5.2. Computational Approaches

An underlying concept to computational efforts in drug repurposing is the signature reversal principle (SRP). This assumes that if a drug can reverse the expression signature of a given set of genes that underlie hallmark(s) for a particular disease, or alter the signature in such a way that it is closer to the “healthy” state, the drug might be effective against the particular disease. The signature in question is often transcriptomic, proteomic, or metabolomic in nature. Despite its simplicity, the application of this principle has led to the repositioning of several drugs as chemo-sensitizers based on anticancer drug-resistant signatures [168]. 

Genome-wide association studies (GWAS) have also led to the identification of novel targets targetable by pre-existing drugs. In situations where pre-existing drugs cannot readily perturb a predicted target, pathway or network mapping has provided a complementary method to these GWAS results. The basic idea is that identifying active signaling pathways or reconstructing genetic, transcriptomic, proteomic, or multi-omic networks associated with the GWAS target may reveal druggable targets.

Finally, concepts from molecular theory have led to the prediction of novel ligand-target binding site complementarity, resulting in several drug repurposing successes in several cancers. The antimalarial drug amodiquine was found to inhibit HSP27 chaperone function and reverse drug-resistance in multiple myeloma cell lines [169]. Similarly, levosimendan, a heart failure drug, was predicted and verified to have anticancer effects based on its binding compatibility and subsequent inhibitory effects of RIOK1 and other kinases [170]. 

### 5.3. Drug-Relevant Databases

The availability of databases containing drug target and phenotypic annotations has vastly increased the possibility of creating novel models and hypotheses for drug mechanisms and applications. As independent components or parts of larger algorithmic workflows, drug-relevant databases greatly facilitate drug-repurposing efforts. Generally speaking, these databases can be organized into four broad categories [171]: (1) chemical, which contains information on drug structure, similarity of structure, mechanisms of action, or drug combination responses (e.g., DrugCombDB [172], CMap [173]); (2) biomolecular, which contain information ranging from genomic annotation to signaling pathway annotations and visualizations (e.g., Cancer Cell Line Encyclopedia—CCLE [174], GenBank, KEGG, CMap [173]; (3) drug–target interactions (e.g., DrugBank [175,176], ChEMBL [177], Drug gene interaction database [178]); and (4) disease association, which contains information such as protein–disease associations (e.g., Open Targets [179], Therapeutic target database [180]). Finally, the integration of electronic health records (EHRs) has been steadily increasing. Machine learning methodologies such as natural language processing, latent semantic indexing, and support vector machine algorithms can analyze and efficiently identify the presence of underlying signals hidden in the diagnostic and pathophysiological data available in EHRs. Ultimately, the ability to draw upon these curated databases and EHRs offers a valuable opportunity to identify hidden patterns and maximize the benefits of drugs currently available for brain tumor therapy.

## 6. Discussion

Despite extensive efforts focused on elucidating the complexities of brain tumor etiology and pathology, the prognosis for brain tumors remains dismal. Multiple genetic and non-genetic factors across length and time scales underlie heterogeneity within and across tumors; each tumor and each patient is unique. Consequently, understanding and addressing tumor complexity requires a multi-level systems approach. Integrating established and emerging experimental methodologies and computational capabilities in a cross-disciplinary environment would enable the comprehensive characterization and analyses of brain tumors. In such a paradigm, multi-omic profiling of matched tumor tissue, blood, and CSF would advance existing systems-scale models of brain tumors and provide mechanistic insights into how genetic and non-genetic mechanisms contribute to tumor progression and enable precision care. Similarly, longitudinal monitoring of patients would provide data into patient-specific treatment effects and would inform on whether treatment modifications are required to maximize the treatment response and reduce the risk of recurrence. 

While we have outlined multiple examples of how systems biology approaches have produced insights into the complexities of brain tumors and will continue to advance our understanding, several challenges remain. There remains a need to develop a more precise, noninvasive assessment of biologic and metabolic features that occur within the tumor, particularly at early stages. Given the sensitive location of brain tumors, improvements in drug delivery are required to overcome the BBB. Both primary brain tumors and BMs disrupt the BBB, which manifest as hypertense regions in contrast-enhancing T1-weighted (T1w) MRI sequences. Although the resulting compromised BBB, referred to as the blood–tumor barrier (BTB), is more permeable than a normal BBB, multiple results have indicated extensive heterogeneity in BBB/BTB integrity [181]. Towards quantifying the extent of disruption, dynamic contrast-enhanced (DCE) MRI has been used to measure transport constants of molecules across different contrast-enhancing regions. The results have shown varying constants throughout those regions, suggesting varying integrity. Indeed, the structural integrity of the BBB/BTB is heterogeneous across medulloblastoma subtypes as well as brain metastases originating from different breast cancer subtypes [182]. Given the varying degree of BBB/BTB integrity, heterogeneous permeability, and heterogeneous perfusion across the BBB/BTB, it is reasonable to assert that the BBB/BTB results in suboptimal drug accumulation of an effective drug to the entire tumor cell population, which contributes to ineffective treatment [181]. For example, cisplatin/etoposide in combination with local irradiation is an effective treatment in approximately 25% of small-cell lung cancer. However, of those tumors that have metastasized to the brain, where neither cisplatin nor etoposide have a significant distribution, this combination has a lower treatment efficacy rate. Anecdotal reports of limited drug distribution in the brain of vemurafenib (ABT-414) also suggest that improved delivery of the drug may benefit some GBM patients [183]. Taken together, clinical evidence indicates that there remains a critical need to overcome the challenges presented by the BBB that impede drug delivery. Finally, the need remains for more accurate models and a deeper understanding of the distribution and dynamics of drugs in the central nervous system to optimize drug selection and delivery, ultimately improving the probability of successful therapy. 

**Figure 3:**
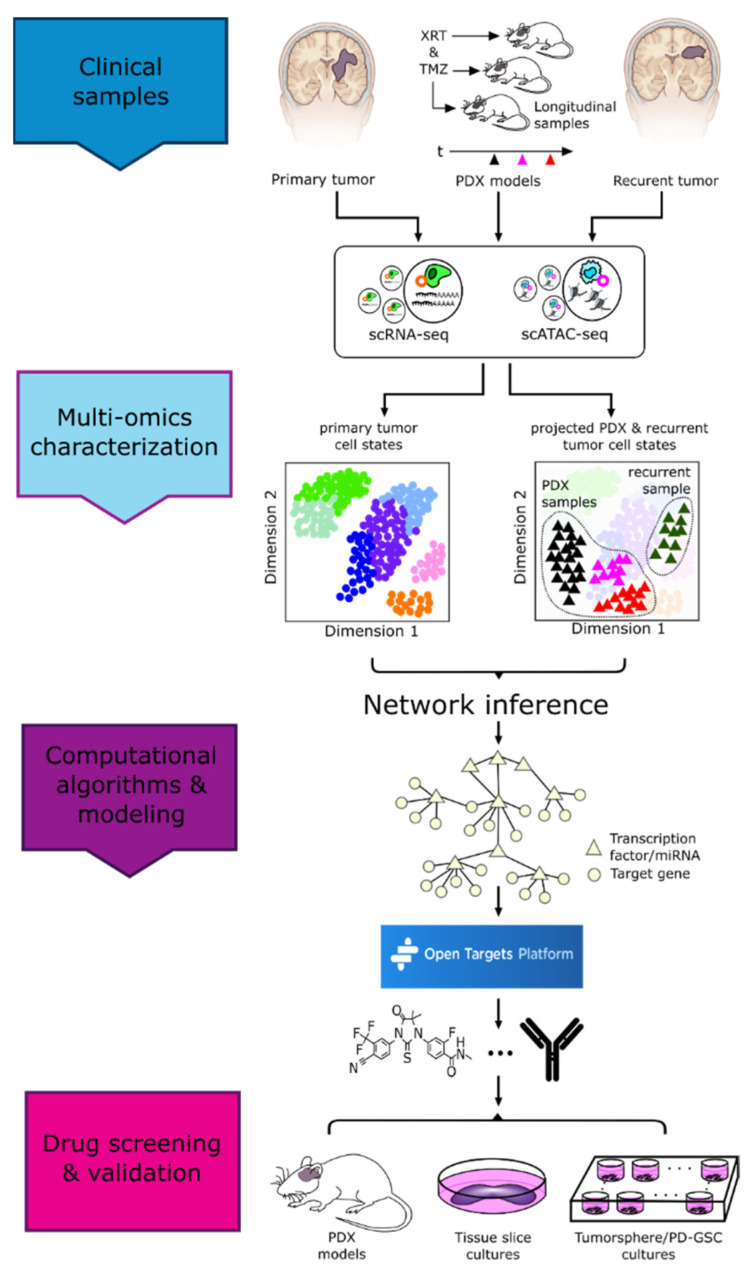
A systems biology framework towards treating brain tumors. The flow diagram illustrates our proof-of-concept work that integrates available technologies towards the implementation of personalized, precision care of brain tumors. We have developed a framework that utilizes several of the technological advances discussed in this review, in which we performed comprehensive characterization and modeling of an individual’s brain tumor and its post-treatment progression. The result was the identification of distinct cell states, defined by their transcriptomic and inferred transcriptional regulatory states. Using this information, we identified putative drugs that target multiple regulators identified from this multi-omic analysis of an individual brain tumor and corresponding PDX models. Ultimately, the results from such an analysis would be tested using various systems including patient-derived cell lines, PDX models (depending on time), and/or tumor slice cultures.

The previous examples of pre-clinical findings and experimental/computational advances highlight the tools available that can drive the realization of precision medicine. Concomitantly, a viable framework is needed to integrate these technologies and apply them in a clinical setting. We posit one such framework based on a multi-institutional collaborative effort among clinical researchers, neurosurgeons, systems biologists, and engineers. Here, we take advantage of the convergence of experimental and computational methodologies currently available, some of which we have developed, towards improving the clinical treatment of GBM patients by providing a rigorous characterization of the regulatory network state of a tumor cell population for an individual patient. As a proof-of-concept, we performed multimodal single-cell profiling (scRNA-seq and scATAC-seq) of a GBM biopsy to characterize the transcriptomic and epigenomic states of tumor cells (Figure 3) [184]. Using corresponding PDX mice, we also modeled how those tumor cells would respond longitudinally to irradiation and chemotherapy with TMZ. The combination of the direct characterization of the tumor biopsy and treated PDX models enabled us to characterize the treatment-induced evolution of tumor cells and identify tumor cell states underlying this progression. Next, by integrating multiple scRNA-seq batches and applying computational pipelines such as SYGNAL [154] and ArchR [151], we inferred regulatory network modules that distinguished subpopulations of tumor cells based on their regulatory network dynamics, informing on putative regulators active during specific periods throughout the course of the disease, and modeled post-treatment progression. Using this information, we then searched through Open Targets [179] to identify pre-existing anti-cancer drugs that putatively target regulators and/or downstream target genes of network modules identified to be active at various stages of tumor progression, e.g., in an adjuvant, or recurrent setting. Although the timing of this proof-of-concept work prevented testing of these drug predictions, ideally, we would have tested these predictions on corresponding tumor tissue slices or in vitro cultures of GSCs or differentiated tumor cells derived from the primary tumor biopsy.

As this framework is a proof-of-concept, we acknowledge that there are opportunities for refinement. For instance, the lead-time in generating PDX models may not be suitable for some patients due to the rapid progression of GBM. Despite this issue, PDX models are still of value for multiple reasons. Beyond the fact that they are patient-specific and capture the effects of tumor architecture, data generated from multiple PDX models from multiple patients would provide valuable insight into what potential tumor-cell states arise post-treatment and what potential targets associated with these states may be manipulated to augment clinical treatment for future patients that may exhibit similar tumor cell subpopulations. Another gap in this framework is the inability of immunocompromised PDX models to capture the effects of immune system interactions on tumor cell evolution. Immunocompetent mouse models do exist, however, and can mimic various aspects of the brain tumor–immune response spectrum [185]. The use of immunocompetent mice as a model system is certainly worth exploring, particularly in regards to investigating immunotherapies [185]. Finally, this framework did not directly characterize the spatial heterogeneity of the patient tumor. However, this approach, as with any other current procedure or methodology, is constrained by sample availability. While characterizing multiple samples across the tumor would improve spatial heterogeneity profiling, that possibility will depend on the various sampling demands for that particular patient tumor biopsy. Some studies have attempted to address these issues [108,186], but systematic characterization of spatial heterogeneity over macroscopic length scales (centimeters) within a tumor are lacking. Novel approaches that make use of autopsy tissue [184,187] may be more successful at addressing these issues as these types of studies are less constrained by tissue availability. If additional tumor slices were available, the use of GeoMx (NanoString^®^) or Visium (10x Genomics) would provide spatial transcriptomic information that would help identify biomarkers or other potentially targetable, spatially distinct, transcriptional phenotypes. Ultimately, integrating single-cell technologies with computational methodologies to infer regulatory network mechanisms, identify tumor-cell states, and predict drug–target combinations for a patient offers an approach that would inform brain tumor treatment on an individual patient level.

## 7. Conclusions

Systems approaches have facilitated the discovery of new insights into the complexity and potential vulnerabilities in brain tumors. While many challenges remain and require a concerted effort across multiple disciplines and institutions, an integrated systems approach can provide immediate benefits to those suffering from brain tumors. Given the improvements in accuracy and precision of HTP technologies and advances in computational and experimental modeling approaches, improving treatment for brain tumors is an achievable goal. Creating an environment where in-depth molecular profiling and longitudinal assessment of a patient’s response to drugs would foster the generation of valuable insight into the molecular underpinnings of drug distribution, tumor response kinetics, and tumor progression. We remain optimistic, encouraged by the continual technological advances made and insights gained about the mechanisms underlying brain tumor structure and pathology, which will ultimately enable longer-term management of these tumor types.
